# Case of Gangrene of the Leg from Phlegmasia Dolens; Case of Tracheotomy for Tumor at the Glottis

**Published:** 1879-06

**Authors:** R. G. Bogue

**Affiliations:** Chicago


### Notes from Private Practice.

### Article VII.

Case of Gangrene of the Leg, from Phlegmasia Dolens; Amputation; Recovery—also, a Case of Tracheotomy for Tumor at the Glottis; Recovery. Reported to the Chicago Medical Society, April 21st, 1879.

I. *Phlegmasia Dolens.*

Mrs. J. married, aged twenty-four years, healthy, delivered Feb. 18th, 1878; first child; labor natural and not severe nor prolonged; remained very comfortable with apprarently nothing out of the way until the 23d, when severe pain began in the calf of the left leg, which steadily increased in severity until it became terrific, necessitating large doses of opiates to give relief, even to the extent of a grain of morphine hypodermically at a time. The leg was swollen but little, and only moderately tender. The pain continued in its severity, without much change in the limb, except moderate swelling and some tenderness behind the knee, until the morning of the 2d of March, when there was noticed a dark colored spot on the ball of the foot and upon the side of the great toe. The severe pain continued and the discoloration and coldness and loss of sensation rapidly extended, so that on the afternoon of the 5th of March the limb was thoroughly in gangrene, from the middle of the leg downward, the whole member but little swollen; the foot a little shrunken. There was tenderness behind the knee and along the course of the femoral vessels, the latter very slight; pulsation of the femoral, just below Poupart's ligament, was quite feeble; the heart beat about eighty times per minute, and intermittent; the long saphena vein was neither distended nor tender.

At this date, the 5th, I first saw the case, with Dr. Hessert, who was in attendance on the case and had been since the evening of the 2d. There was very little or no odor from the limb. It was well wrapped in flannel, wet with a solution of alcohol, covered by rubber cloth, and surrounded by bags of hot bran or hot sand. There was given 0.25 of salicin four times a day, and opium sufficient to relieve the pain. She was encouraged to take milk, soup, coffee, eggs and any other food she desired, and a moderate amount of stimulant. By the 7th the gangrene had extended to within about five inches of the knee, the leg above the gangrene somewhat inflamed, the thigh swollen and a red tender line along the track of the lymphatics. There was rather less pain, or it was not so severe continuously, yet there were spells of severe pain. The patient was eating fairly, but felt a good deal depressed. There had been not more than the usual amount of lochia; it had been offensive, but had now ceased. She had at no time complained of pain, or discomfort even, in the lower part of the abdomen or pelvis. The patient continued in about this condition, really not losing ground and the gangrene not extending, suffering less pain, pulse becoming steady and ranging from one hundred and twelve to one hundred and twenty per minute, up to the 14th, when there was less inflammation of the thigh and upper portion of the leg, with a line of demarcation beginning to form; there was a good deal of odor now from the limb. Amputation was now advised as soon as the line of demarcation should be fairly established.

On the morning of the 16th, with the assistance of Drs. Hessert, Hooper, Bert and Hann, I amputated the limb at the knee joint. There was not room to make a stump below this point and further I believed disarticulation a safer operation than amputation either just below or just above the joint. The thigh was quite œdematous, but soft. The soft parts at the point of operation, were quite firm, from infiltration with sero-plastic effusion. Lateral flaps, according to Stephen Smith's plan, were made, brought together and secured by three or four sutures in the anterior two-thirds, the posterior portion being left open so as to secure free drainage. The thigh was supported by a many-tailed bandage, and the stump dressed with carbolated water, and rested upon a small pillow, which was afterward changed to an air pillow. The flaps were composed of skin and subcutaneous connective tissue, the straight part of the incision posteriorly extending upward to a point squarely behind the joint, the soft tissues in the popliteal space were divided at about the middle of the space, leaving barely the upper parts of the heads of the gastrocnemius.

At the point of division of the popliteal vessels the artery was patulous, but small; it was unobstructed as far downward as examined, which was beyond where it breaks up; it must have been unobstructed upward, for it throbbed after ligation as any large artery is wont to do in the end of a stump. But the two popliteal veins—there were two—and also all of the deep veins, were filled with firm clot, the subcutaneous ones being free. All of the veins in the popliteal space were plugged. In other words the whole system of deep veins of the leg was filled with thrombi, while the superficial one was not. How far upward there was thrombus of the deep veins of the thigh cannot be said, but certainly not above where the internal saphena joins, for this was unobstructed. The patient recovered from the operation and progressed slowly to recovery. There was some burrowing of pus in the lower part of the thigh, which necessitated opening for drainage and cleansing. About two weeks after the operation there came on a phlegmasia dolens of the other limb; it was mild and gave but little pain, yet there was tenderness and hardness along the track of the femoral vessels. It served to pretty thoroughly frighten the patient and friends.

The diagnosis at first was gangrene from embolism of the femoral artery. As the gangrene occurred rather suddenly, no pulsation was to be felt in the leg nor popliteal region, and but feebly in the common and first portions of the superficial femoral and the saphenous vein was not obstructed; but it proved to be a case of gangrene from extensive occlusion of the veins; from that demonstrated condition of the veins, phlegmasia dolens implicating in this case the deep ones, leaving the superficial ones comparatively free. I judge that this result of the affection must be very rare, for I have found only bare mention made of it by Dr. Fordyce Barker, in his book on The Puerperal Diseases, saying that he had never seen a case, but that cases had been reported.

It would seem that gangrene occurred in this case as a consequence of the sudden obliteration of a large portion of the veins of the limb, thereby depriving the limb of sufficient capillary circulation to sustain it. And as there was not adequate means of returning the usual amount of blood from the limb, there was only a moderate amount sent to it, as shown by the feeble pulsation of the common femoral of that side as compared with the other.

2. *Tumor of Glottis; Tracheotomy.*

Willie Young, aged about 10 years; in July, 1875, began to cough, as of ordinary cold, which persisted with some expectoration of mucus. Cough became more and more troublesome and harsh; the breathing became noisy and at times a little difficult, especially when sleeping. He gradually lost flesh and steadily these evidences of interference with respiration referable to the larynx, became more pronounced.

In November when I saw him, respiration was performed with some effort, becoming more difficult, when sleeping. Patient was emaciated, coughed and expectorated a good deal. Dr. H. A. Johnson saw him, and upon laryngoscopic examination there was found to be general chronic inflammation of the lower part of the pharynx, ulceration of the top of the epiglottis, and in the left aryteno-epiglottic fold a small tumor which folded that side of the epiglottis downward and toward the middle line, itself falling upon the glottic opening. The tumor, and general inflammatory swelling of the glottis encroached upon the opening so much that respiration was difficult, and especially so when the laryngeal muscles were off their guard during sleep or when there was spasm of the larynx, as occasionally happened. It was decided to apply to the parts by means of the hand atomizer a 4 per cent. solution of sulphate of zinc every second or third day, using once or twice a day a gargle of alum. Quinine was given, 0.15 morning and evening, and the taking of nourishing food was encouraged. All of this was to be tried, being ready to perform tracheotomy at any time in case of emergency. Instead of improvement, the respiration became more and more difficult, so that it was deemed best to resort to the operation, not waiting for emergency.

On January 2d, 1876, with the assistance of Drs. Johnson and John Bartlett, I operated; gave ether; opened the trachea and introduced a tube, after which he breathed perfectly easy, and slept quietly. There was a good deal of discharge of mucus through the tube from the bronchitis. The quinine was continued and the sulphate of zinc applied every other day to the parts by means of a pharyngeal or post-nasal syringe. The boy began to sleep well, eat well, breathe without any difficulty and regain his flesh. The general inflammation of the pharynx subsided, ulcer of the epiglottis healed, but the tumor remained red and about the same in size for a long time. In May he had an attack of whooping cough, for which I gave him pretty large doses of bromide of potassium and laurel water, which was continued for a couple of weeks or more. In the meantime the tumor had sensibly diminished in size. Iodide of potassium was combined with the bromide and given for about two months; the tumor gradually became smaller and the surface light-colored. From the disappearance of most of the tumor and all of the inflammatory thickening, the opening to the larynx became quite free, and the boy could breathe very easily with the inner tube removed and the outer one plugged. In this way he respired through the larynx most of the day and through the tube at night. About the middle of October I removed the tube. At this time there was no obstruction, the tumor had shrunken away to a small nodule; the ulcer had healed, leaving some deformity of the epiglottis, the boy in good health, but had partial loss of voice. The opening in the neck closed within a few day after removal of the tube. He has had no return of laryngeal difficulty nor has he regained his voice.

Now, at the end of three years from the time of the operation the boy is well, having only a little harshness of respiration and able to speak in a tone about one-half between a good whisper and full voice. As to the nature of the tumor, I can give no decided opinion; its disappearance under the use of potassium would suggest a specific cause. Ulceration in that locality, probably implicating somewhat the vocal cords, although at no time was ulcer of them seen, would be suspicious. There are not apparent evidences of hereditary specific disease.

R. G. BOGUE, M.D.

CHICAGO.

---

IN THE GLASGOW DISPENSARY FOR SKIN DISEASES, a page in brilliant costume directs the patients to the several apartments, and a gentleman connected with one of the fashionable pharmacies of the city, dispenses the medicines ordered, which are supposed to be paid for. Will the Central Free Dispensary of Chicago suffer itself to be outdone by any such bloated institution in the "effete monarchies of Europe?"

WE are under obligations to Dr. Geo. H. Cushing, one of the Publication Committee of the American Dental Association, for plate and copy of the original and interesting report on Histology and Microscopy, by Dr. G. F. Waters, published in another column.

